# Clinical Manifestation of a Calyceal Diverticular Abscess in a Pregnant Woman

**DOI:** 10.1155/2014/975071

**Published:** 2014-11-30

**Authors:** Masaki Sekiguchi, Yuichi Hasegawa, Satoko Kinomoto, Haruhiko Sago

**Affiliations:** ^1^Center for Maternal-Fetal-Neonatal and Reproductive Medicine, National Center for Child Health and Development, 2-10-1 Okura, Setagaya-ku, Tokyo 157-8535, Japan; ^2^Division of Urology, Department of Surgical Subspecialties, National Center for Child Health and Development, 2-10-1 Okura, Setagaya-ku, Tokyo 157-8535, Japan

## Abstract

Calyceal diverticula are congenital, nonsecretory abnormalities in which the transitional cell-lined cavity communicates with the renal collecting system. Here we present the case of a calyceal diverticular abscess during pregnancy. A 40-year-old primiparous woman developed the abscess at 23 weeks of gestation, with right flank pain and a 37.8°C fever. A transabdominal ultrasound revealed a 12 × 10 cm cystic mass in the right kidney. She was initially diagnosed with a simple renal cyst infection, and intravenous antibiotics were initiated. Percutaneous drainage was started at 26 weeks of gestation. When urine excretion from the cyst was confirmed by dye test using indigotindisulfonate sodium, the patient was diagnosed with a calyceal diverticular abscess. She gave birth to a 2,870 g healthy male at 38 weeks of gestation. Percutaneous drainage with low-dose antimicrobial therapy could thus allow for the continued pregnancy of women with a calyceal diverticular abscess until full term.

## 1. Introduction

Calyceal diverticula are congenital, nonsecretory abnormalities in which the transitional cell-lined cavity within the renal parenchyma communicates with the collecting system through a narrow infundibulum [1]. Its prevalence is 0.21–0.4% of the general population [2, 3]. Calyceal diverticula can cause pain, hematuria, urinary tract infection, and damage to surrounding renal parenchyma due to compression and may require surgical intervention [1].

Only one report to date has addressed pregnancy complicated by a calyceal diverticular abscess [4], and management is therefore not well established. In this report, we describe the clinical course of a pregnant woman with a calyceal diverticular abscess that was first diagnosed as a simple renal cyst infection.

## 2. Case Presentation

The patient was a 40-year-old Japanese woman in her first pregnancy, who conceived by intracytoplasmic sperm injection. A renal cyst of 4 cm in diameter had been found at 36 years of age, but there was otherwise no past history of urinary tract infection or other remarkable family history.

**Figure 1:**
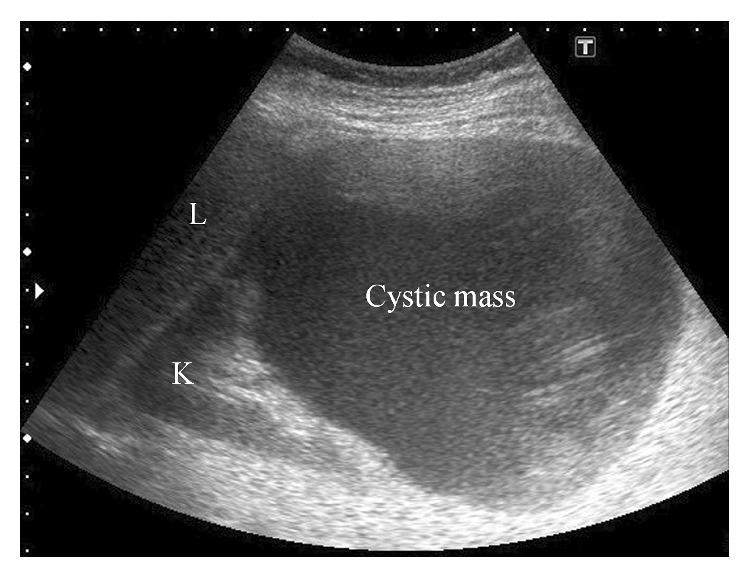
Ultrasonographic image of the right kidney, demonstrating a 12 × 10 cm cystic mass in the lower pole of the right kidney. L, liver; K, right kidney.

The patient was referred to our hospital at 8 6/7 weeks of gestation and started prenatal care in our outpatient clinic. At 23 5/7 weeks of gestation, she presented with a 37.8°C fever and pain in her right flank. Physical examination found right costovertebral angle tenderness, and transabdominal ultrasonography (USG) revealed a 12 × 10 cm cystic mass in the lower pole of the right kidney (Figure 1). Because laboratory tests showed leukocytosis (15.06 × 10^9^/L), elevated serum C-reactive protein (CRP, 75 mg/L), and low white blood cell (WBC) count in the urine (5–9/HPF), she was diagnosed with a simple renal cyst infection. The patient was admitted and given an intravenous injection of ampicillin/sulbactam (1.5 g, three times a day). Her pain and fever resolved the second day after admission, and inflammation gradually decreased (8.90 × 10^9^/L WBC and 34 mg/L CRP on the third day; 7.74 × 10^9^/L WBC and 4 mg/L CRP on the ninth day).

**Figure 2:**
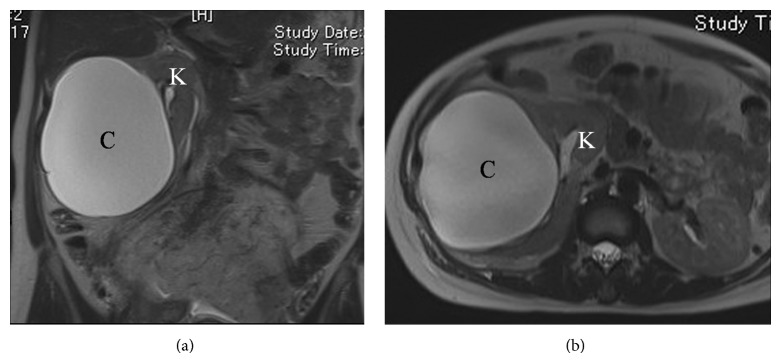
Magnetic resonance imaging (T2-weighted image) of a large cystic mass arising from the lower pole of the right kidney. (a) Coronal and (b) axial view. C, cystic mass; K, right kidney.

The patient underwent magnetic resonance imaging (MRI) at 24 6/7 weeks of gestation to investigate the cyst in detail. The MRI revealed a large cystic mass arising from the lower pole of the right kidney, as shown in the transabdominal USG, that was compatible with the primary diagnosis of a simple renal cyst (Figure 2).

Although the infection resolved with antibiotic therapy, the size of the cyst was unchanged. We decided to puncture the cyst to reduce the risk of recurrent infection or rupture, as the uterus in late pregnancy would be more enlarged and compress the urinary tract. At 26 4/7 weeks of gestation, percutaneous puncture was performed under ultrasound guidance to drain 800 mL of culture-negative, pus-like fluid, and the cyst was deflated. Drainage continued at 600–800 mL/day from a nephrostomy catheter left in the cyst. A dye test was performed by systemic intravenous injection of indigotindisulfonate sodium (Indigo Carmine) to examine communication between the cyst and the renal collecting system. We found dyed urine excretion from the cyst, which confirmed the cyst as the calyceal diverticulum. The patient was discharged on day 10 after nephrostomy and was started on oral cefaclor (0.25 g/day) prophylactically. Serial examinations showed no recurrence of the calyceal diverticular abscess until delivery.

**Figure 3:**
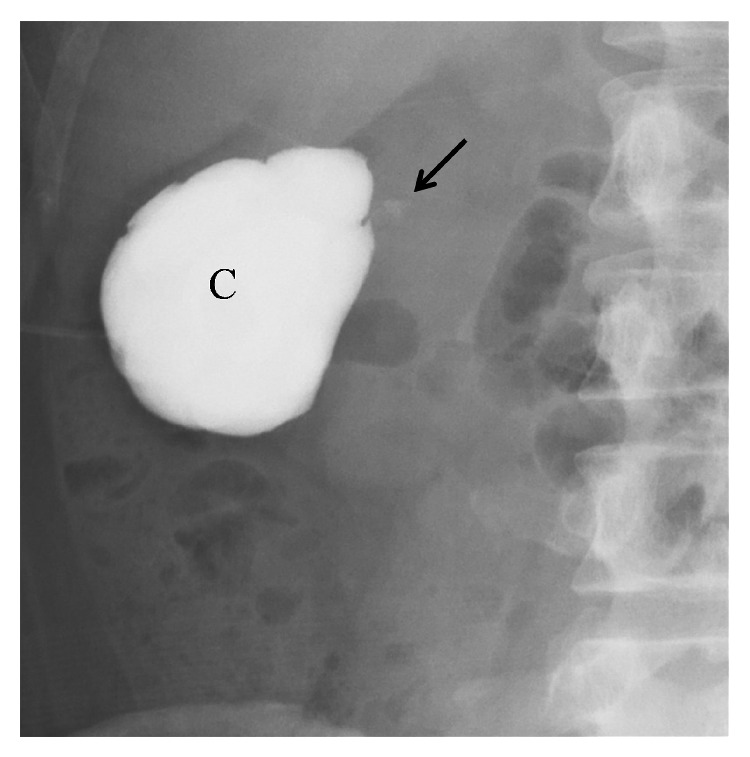
Anterograde pyelography demonstrating communication between the cystic mass and the collecting system (arrow). C, cystic mass.

The patient gave birth at 38 4/7 weeks of gestation to a 2,870 g healthy male, with Apgar scores of 8 (at 1 minute) and 9 (at 5 minutes), by vacuum extraction because of arrested descent in the second stage of labor. Her postpartum period was uneventful. Anterograde pyelography on day 4 after delivery revealed communication between the cyst and the collecting system, and the cyst was clinically confirmed to be a calyceal diverticulum (Figure 3). The patient was discharged on day 6 after delivery, with a nephrostomy catheter left in the diverticulum. Surgical intervention is under consideration.

## 3. Discussion

The present report described the successful pregnancy of a patient with a calyceal diverticular abscess. We could not initially give a precise diagnosis because imaging tools were restricted to USG and MRI, which have no adverse effects on the fetus.

Identifying communication between the cyst and the renal collecting system is essential in distinguishing a calyceal diverticulum from a simple cyst. However, radiographic examinations with contrast agents are contraindicated during pregnancy due to fetal side effects. In this case report, intravenous indigotindisulfonate sodium injection was used as a proxy for the radiographic dye test because it is relatively safe for the fetus (FDA risk category of B). This agent helped detect urine secretion from the cyst, indicating communication between the cyst and the collecting system.

Asymptomatic calyceal diverticula generally do not need treatment. However, enlarged calyceal diverticula with infection or pain often require intervention such as surgical resection [1, 5]. As the safety of surgical intervention during pregnancy was unclear, we chose a less invasive option (i.e., transpercutaneous drainage) that was previously reported [4].

Although we initiated antimicrobial therapy before drainage, infected renal cysts and/or large renal abscesses (e.g., >5 cm in diameter) are often refractory to antibiotics [6, 7]. We controlled symptoms of infection by initiating antibiotics before drainage in the present case; however, if the antibiotics were not effective, we would have initiated transpercutaneous drainage.

Transient fever due to temporary bacteremia during percutaneous drainage is a common complication, while gastrointestinal tract injury, significant bleeding, pneumothorax, and urinary tract injury are rare but more critical [8, 9]. Concurrent antimicrobial therapy and USG guidance are recommended to reduce the risk of complications when performing percutaneous drainage.

The relationship of pregnancy to the onset of calyceal diverticular abscesses is unclear. Primary onset of the abscess in the present case was at late pregnancy (at 24 weeks of gestation), which was similar to that of a previously reported case (at 28 weeks of gestation) [4], suggesting that compression of the urinary tract by an enlarged uterus might enlarge and infect the calyceal diverticulum. Increasing levels of progesterone in the late second to the third trimester may play a role in the infection with its bladder-relaxing effect leading to increased volume of residual urine and vesicoureteral reflux.

In summary, we could successfully manage the pregnancy complicated by the calyceal diverticular abscess with antimicrobial therapy and percutaneous drainage, and the dye test using indigotindisulfonate sodium was helpful for making the diagnosis without radiographic examinations using contrast agents.
